# Percutaneous Mitral Valve Repair in Mitral Regurgitation Reduces Cell-Free Hemoglobin and Improves Endothelial Function

**DOI:** 10.1371/journal.pone.0151203

**Published:** 2016-03-17

**Authors:** Christos Rammos, Tobias Zeus, Jan Balzer, Laura Kubatz, Ulrike B. Hendgen-Cotta, Verena Veulemans, Katharina Hellhammer, Matthias Totzeck, Peter Luedike, Malte Kelm, Tienush Rassaf

**Affiliations:** University Hospital Düsseldorf, Medical Faculty, Division of Cardiology, Pulmonology and Vascular Medicine, Moorenstrasse 5, 40225, Düsseldorf, Germany; University Francisco de Vitoria School of Medicine, SPAIN

## Abstract

**Background and Objective:**

Endothelial dysfunction is predictive for cardiovascular events and may be caused by decreased bioavailability of nitric oxide (NO). NO is scavenged by cell-free hemoglobin with reduction of bioavailable NO up to 70% subsequently deteriorating vascular function. While patients with mitral regurgitation (MR) suffer from an impaired prognosis, mechanisms relating to coexistent vascular dysfunctions have not been described yet. Therapy of MR using a percutaneous mitral valve repair (PMVR) approach has been shown to lead to significant clinical benefits. We here sought to investigate the role of endothelial function in MR and the potential impact of PMVR.

**Methods and Results:**

Twenty-seven patients with moderate-to-severe MR treated with the MitraClip® device were enrolled in an open-label single-center observational study. Patients underwent clinical assessment, conventional echocardiography, and determination of endothelial function by measuring flow-mediated dilation (FMD) of the brachial artery using high-resolution ultrasound at baseline and at 3-month follow-up. Patients with MR demonstrated decompartmentalized hemoglobin and reduced endothelial function (cell-free plasma hemoglobin in heme 28.9±3.8 μM, FMD 3.9±0.9%). Three months post-procedure, PMVR improved ejection fraction (from 41±3% to 46±3%, p = 0.03) and NYHA functional class (from 3.0±0.1 to 1.9±1.7, p<0.001). PMVR was associated with a decrease in cell free plasma hemoglobin (22.3±2.4 μM, p = 0.02) and improved endothelial functions (FMD 4.8±1.0%, p<0.0001).

**Conclusion:**

We demonstrate here that plasma from patients with MR contains significant amounts of cell-free hemoglobin, which is accompanied by endothelial dysfunction. PMVR therapy is associated with an improved hemoglobin decompartmentalization and vascular function.

## Introduction

An inappropriate regulation of the vascular tone is regarded as one of the earliest phenomena in cardiovascular disease (CVD). This endothelial vasodilator dysfunction is implicated in the development, pathogenesis and progression of HF [1–3]. It is further identified as prognostic relevant in patients with cardiovascular risk factors or established CVD [4–6]. Given the pivotal role of nitric oxide (NO) in mediating endothelial function, declines in NO bioavailability perpetuate endothelial dysfunction. Notably, NO is not only decreased due to diminished NO synthase (NOS) activity, altered levels of asymmetric dimethyl arginine and L-arginine but also by increased scavenging through decompartmentalized hemoglobin (Hb). Of note, NO is scavenged at least 6,000 times more rapidly by cell-free Hb than by by hemoglobin compartimentalized in red blood cells. This major systemic effect on NO bioavailability by hemolysis-mediated Hb decompartmentalization contributes to impaired endothelial function in a variety of diseases [7]. Mechanistically, such subclinical hemolysis leads to Hb scavenging of NO, which has been shown to abrogate vascular functions during hemodialysis or in sickle cell anemia [8, 9].

Chronic severe mitral regurgitation (MR) leads to a chronic volume overload, hemodynamic and ventricular remodeling. This ultimatively leads to heart failure (HF), pulmonary hypertension and death. MR thus critically influences morbidity and mortality [10, 11]. Reduction of MR severity by percutaneous mitral valve repair (PMVR) with the MitraClip® system has emerged as an option for high-risk patients, otherwise unsuitable for conventional surgery [12–14]. This interventional approach uses the Alfieri's edge-to-edge mitral valve repair technique to reduce mitral valve regurgitation by anterior and posterior leaflet grasping. PMVR reduces the severity of MR, decreases LV dimensions, improves cardiac output and leads to significant HF-related clinical benefits [15]. Improvements after PMVR have been attributed to favorable hemodynamics and loading conditions [15, 16].

Whether the decompartmentalization of Hb contributes to impaired endothelial function in MR patients is not known. We therefore hypothesized that MR increases cell-free Hb, which blunts endothelial function by NO scavenging and that PMVR reduces cell-free Hb, improving vascular function.

## Methods

### Study population and study design

This was a prospective, single-center, and observational study. A total of n = 27 patients with symptomatic severe or moderate-to-severe MR who underwent PMVR with the MitraClip® system (Abbott vascular, Wetzlar, Germany) were included. Prior to PMVR procedure, patients were declined for conventional surgical treatment due to high operative risk by a dedicated heart team decision. All patients gave written informed consent, and study procedures were in accordance with the Declaration of Helsinki. The institutional Ethics Committee of the Heinrich-Heine University approved the study protocol (NCT02033811). Patients’ baseline characteristics are given in Table 1.

**Table 1:** Basic clinical and biochemical characteristics.

		n = 27
	Age (y)	77,4 ± 7,8
	Gender m (n)	18
	Height (cm)	171,3 ± 9,3
	Weight (kg)	77,7 ± 10,1
	BMI (kg/m^2^)	26,6 ± 3,6
	Smoker (n)	3
	NYHA 3 (n)	16
	NYHA 4 (n)	6
	Logistic Euro-Score	23,4 ± 14,2
	Eurosscore II	6,8 ± 5,8
	Primary MR	1
	Secondary MR	26
**Comorbidities**		
	Diabetes (n)	8
	Hypertension (n)	27
	Pulmonary disease (n)	6
	Peripheral artery disease (n)	6
	pHT (n)	14
	CAD (n)	20
	Persistent atrial fibrillation (n)	17
	Hyperlipoproteinemia (n)	14
	CABG (n)	12
	Pacemaker (n)	11
**Previous Interventions**		
	History of CABG (n)	12
	History of valv. intervention (n)	6
	History of AMI (n)	4
**Medications**		
	ACE-I/ARB (n)	12
	Aldosterone-Antagonist (n)	10
	Beta blocker (n)	27
	Diuretics (n)	26
	Anticoagulation (n)	24
**Clinical routine**		
	GFR (ml/min)	46,2 ± 19,2
** **	Hb (g/dl)	11,7 ± 2,2
	Trop (mg/dl)	37,9 ± 23,2
	BNP (pg/ml)	4830,8 ± 5609,8
	CRP (mg/dl)	1,2 ± 1,9
	Total Protein (g/dl)	6,8 ± 0,7

Collected data included patient characteristics, peri-procedural in hospital data, laboratory results and follow-up data up to three month after PMVR. Clinical outcome parameters for follow up after three months were functional NYHA classification.

Prior to the procedure, at pre-discharge and three months afterwards, examinations were performed with clinical assessment, conventional echocardiography and determination of endothelial function. Brachial blood pressure (BP) was measured in duplicate by cuff and mercury sphygmomanometer and the average of the 2 measurements was recorded. Blood was drawn for clinical routine. The Institute of Clinical Chemistry and Laboratory Diagnostics, University Hospital Duesseldorf performed all analyses unless noted otherwise.

### Measurement of cell-free plasma hemoglobin

Cell-free plasma Hb concentrations were determined using the QuantiChrom™ Hb Assay Kit (BioAssaySystems, Hayward, California), as described [8].

### Endothelial Function

Flow-mediated vasodilation (FMD) of the brachial artery as a measure of NO bioaviability was measured by a noninvasive technique to assess endothelial function as described [17–20]. Briefly, after a 15-min equilibration period, with the use of a 12-MHz linear-array transducer (Vivid I, GE Healthcare, Germany), the brachial artery diameter and Doppler-flow velocity were acquired proximal of the antecubital fossa before and immediately after cuff deflation of 5-minute forearm arterial occlusion at 200 mmHg of pressure. End-diastolic frames were obtained and analyzed with an automated analysis system (Brachial Analyzer, Medical Imaging Applications, Iowa City, IO) by blinded investigators. Nitroglycerin-mediated vasodilation (NMD) i.e., endothelium-independent vasodilation was measured at 4 min after 400 μg sublingual nitroglycerin in patients with adequate blood pressure [21–23]. FMD was determined as the maximal percent diameter change of the arterial diameter measurement relative to the baseline measurement. Further, FMD values of MR patients were compared to healthy age-matched controls and age-matched high-risk controls, in patients with coronary artery and end stage renal disease [19, 24]. Characteristics for these subgroups are given as supplementary material (S1 Table).

### MitraClip® system

The MitraClip® procedure was performed either in general anesthesia or deep sedation, as described [25–27]. The detailed procedure has been described before [13, 14]. In brief, with the guidance of fluoroscopy and transesophageal echocardiography, transseptal puncture was performed. The transseptal sheath was exchanged for the steerable guide catheter and dilator. The Clip Delivery System (CDS) was introduced into the guide catheter and the MitraClip^®^ device advanced into the left atrium. The CDS was brought to the mitral valve, and leaflets were grasped and the clip deployed.

### Echocardiographic imaging and analysis

Transthoracic echocardiography was performed with commercially available standard ultrasound scanners (Vivid 7; GE Healthcare, Germany) with a 2.5-MHz transducer, as described [28, 29]. Classical 2D echocardiographic parameters included end-diastolic left ventricular (LV) diameters. LV ejection fraction (EF) was calculated by using the biplane Simpson method from the apical four- and two-chamber views. All echocardiographic parameters were measured in accordance with the latest European guidelines [30].

### Statistical methods

Results are expressed as mean±standard error (SEM) unless stated otherwise. Differences between baseline and follow-up data were compared using paired Student’s two-tailed *t*-test. p values of less than 0.05 were regarded statistically significant. All statistical tests were conducted using Prism 5.0 (GraphPad) for Mac OS.

## Results

### Baseline and procedural characteristics

Successful clip implantation was performed in all 27 patients. Basic clinical, biochemical, and procedural characteristics are shown in Table 1 and Table 2, respectively.

**Table 2:** Procedural Characteristics.

Procedure duration (min)	117,9 ± 51,2
Radiation dose (cGy*cm^2^)	8859,3 ± 7593,3
Radiation time (min)	31,5 ± 20,8
MitraClip per procedure (n)	1,1 ± 0,3
General anesthesia (n)	9
Deep sedation (n)	18

### Improved LV function and functional class following PMVR

**Fig 1:**
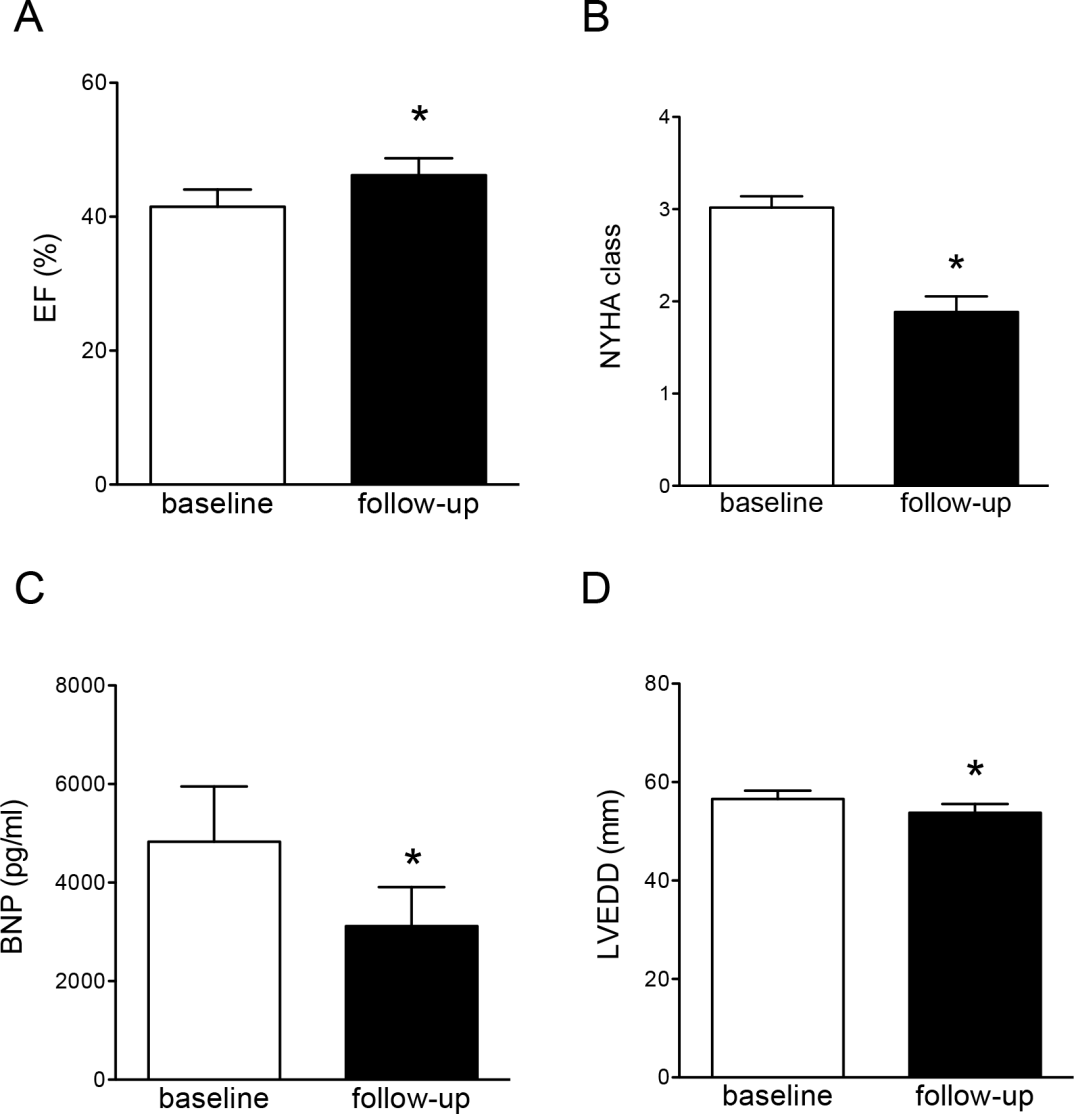
Improved LV functions, global remodeling, and functional class following PMVR. (A) and (B) Improved LV ejection fraction (EF) and NYHA functional class after 3 months follow-up period. (C) Reductions in plasma BNP levels following PMVR. (D) Reversed remodeling indicated by reductions in LV end-diastolic diameter (LVEDD). Data are mean±SEM, * denotes p<0.05, n = 27.

The PMVR procedure led to enhanced LV ejection fraction (EF) and improved clinical status, as assessed by NYHA functional class in the 3 months follow-up period (Fig 1A and 1B). Improvements in LV function and hemodynamics were evidenced by reductions in plasma BNP levels (Fig 1C). Reversed remodeling was further indicated by reductions in LV end-diastolic diameter (LVEDD) (Fig 1D).

### Enhanced endothelial functions and reduced decompartmentalized hemoglobin

**Fig 2:**
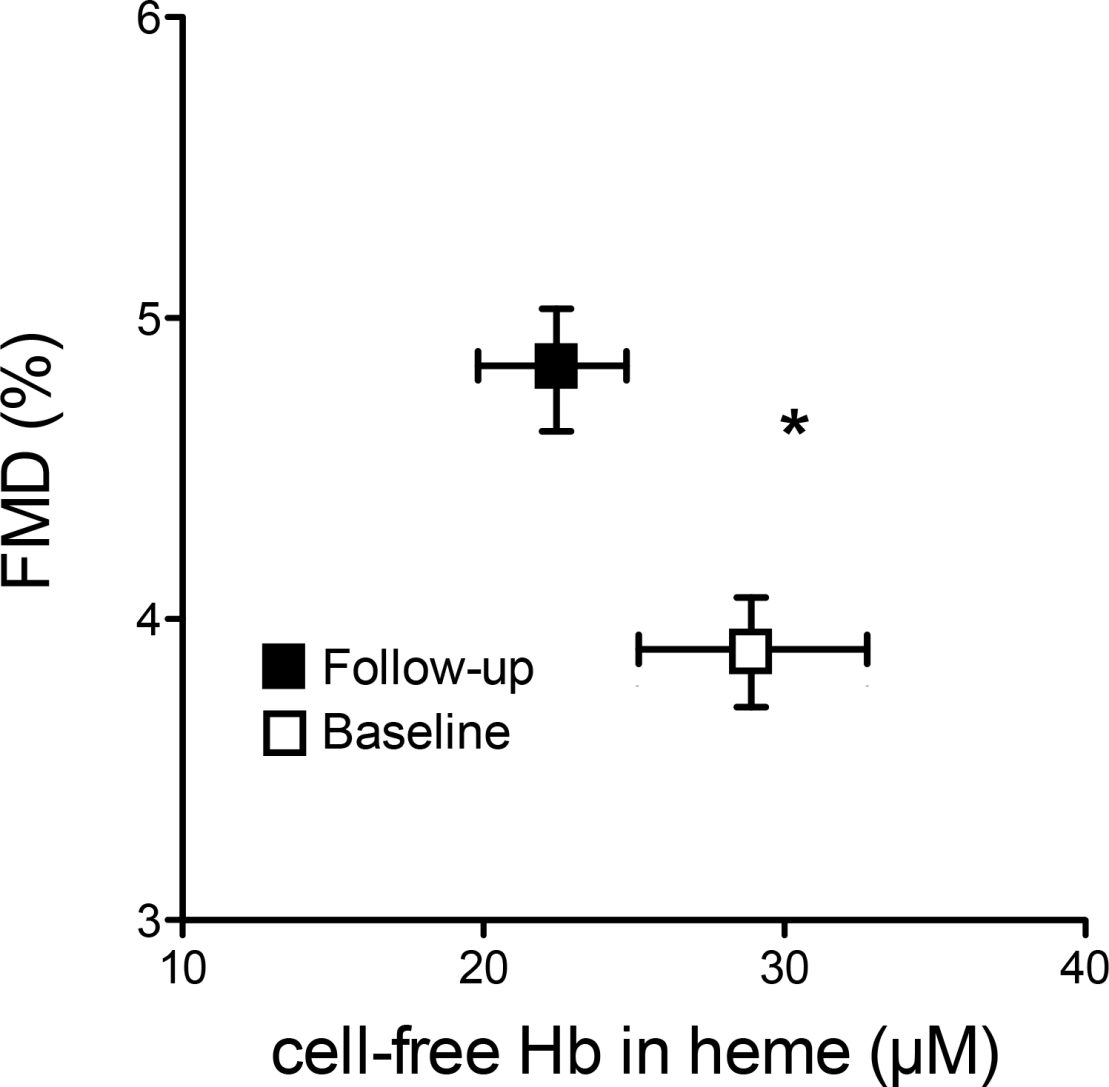
Decompartmentalization of hemoglobin is associated with endothelial dysfunction in MR and improved following PMVR. Cell-free plasma hemoglobin (cell-free Hb) increases and flow-mediated dilation (FMD) significantly decreases following PMVR. Baseline data are given in black, three month follow-up in red. Data given as mean±SD, n = 27. *Each p <0.05.

Baseline levels showed an impaired endothelial function as determined by FMD (3.9±0.9%), which was associated with high levels of decompartmentalized Hb (cell-free Hb in heme 28.9±3.8 μM). 3-months after PMVR the amount of decompartmentalized Hb was reduced (cell-free Hb in heme 22.3±2.4 μM, p = 0.02) and endothelial function was improved (FMD 4.8±1.0%, p<0.0001) (Fig 2 and Table 3).

**Table 3:** Vascular function and blood parameters following PMVR.

	Baseline	Follow-Up	P-Value
**Hemodynamics**			
MAP (mmHg)	94 ± 9	94 ± 8	0.8
Heart rate (bpm)	67 ± 9	68 ± 9	0.9
**Vascular function**			
Diameter BA (mm)	4.5 ± 0.6	4.4 ± 0.5	0.5
FMD (%)	3.9 ± 0.9	4.8 ± 1.0	< 0.001
**Blood parameters**			
Serum-Creatinine (mg/dl)	1.5 ± 0.7	1.5 ± 1.0	0.9
Blood Urea nitrogen (mg/dl)	28.9 ± 15.4	30.7 ± 16.2	0.6
Potassium (mM/l)	4.1 ± 0.6	4.1 ± 0.5	0.7
Calcium (mmol/l)	2.3 ± 0.1	2.3 ± 0.1	0.9
Hb (mg/dl)	11.7 ± 2.3	11.7 ± 1.8	0.7
Cell-free Hb in heme (μM)	28.9 ± 3.8	22.3 ± 2.4	0.02
Hematocrit (%)	36.1 ± 6.1	36.4 ± 4.8	0.8
Haptoglobin (g/l)	1.4 ± 0.5	1.4 ± 0.5	1.0
Total-bilirubin (mg/dl)	0.8 ± 0.5	0.6 ± 0.4	0.01
Lactate dehydrogenase (mg/dl)	242.7 ± 73.4	224.4 ± 41.4	0.3
Iron (μM/l)	67.0 ± 43.0	98.2 ± 145.3	0.3
Ferritin (μg/l)	91.0 ± 76.1	84.5 ± 100.1	0.6
Transferrin (mg/l)	275.3 ± 78.3	280.4 ± 71.4	0.9
Transferrin saturation (%)	18.0 ± 13.0	19.2 ± 16.2	0.9
Soluble transferrin receptor (mg/l)	2.0 ± 0.9	2.0 ± 1.0	0.7

MAP = mean arterial pressure; bpm = beats per minute; BA **=** brachial artery; FMD **=** flow-mediated dilation; Hb **=** hemoglobin

### No impact on endothelial functions and decompartmentalized hemoglobin at pre-discharge

**Fig 3:**
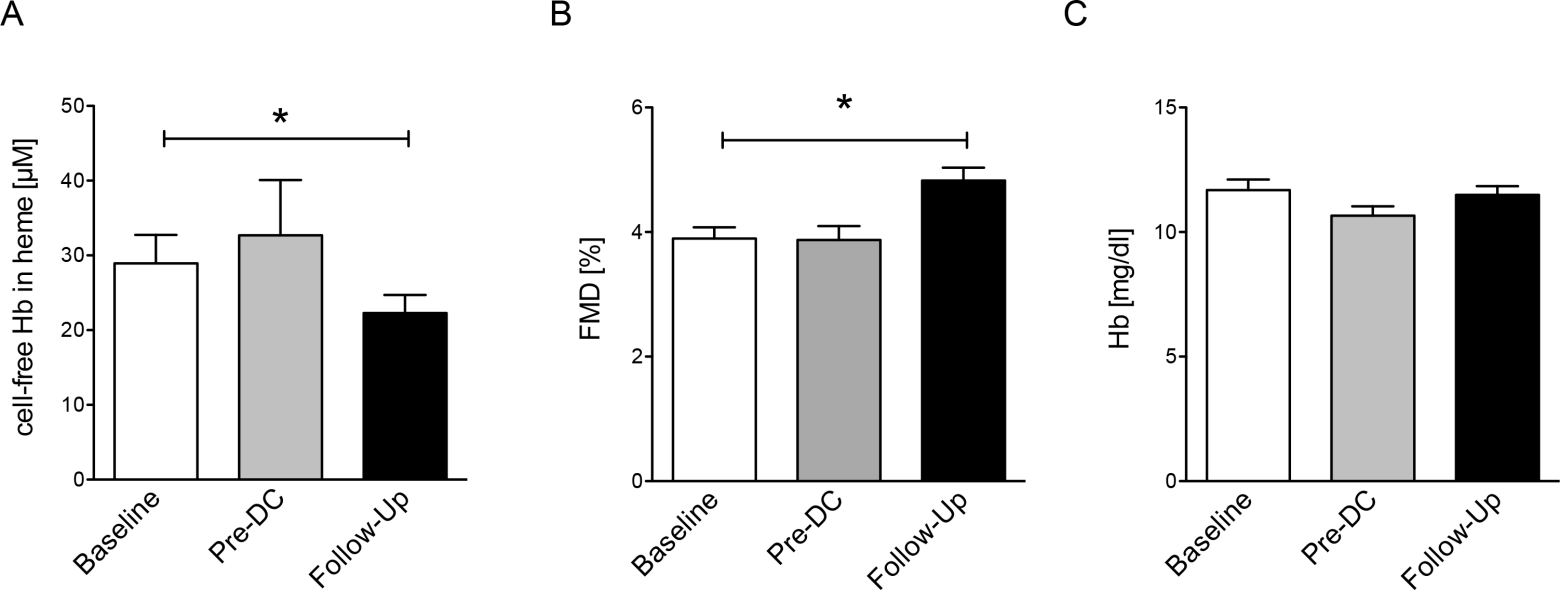
Cell-free plasma hemoglobin, flow-mediated dilation (FMD) and hemoglobin. (A) Reduction of cell-free plasma hemoglobin (cell-free Hb) is observed 3 months following PMVR. (B) Increased endothelial functions after PMVR. (C) No change is observed for Hb. Data given as mean±SD, n = 27. * denotes p <0.05.

To exclude an acute effect of PMVR on endothelial functions through NO scavenging by decompartmentalized Hb we determined cell-free Hb and FMD at pre-discharge, in detail 4–6 days after the PMVR procedure. No effects were observed for cell-free Hb, FMD or Hb (Fig 3A–3C).

**Fig 4:**
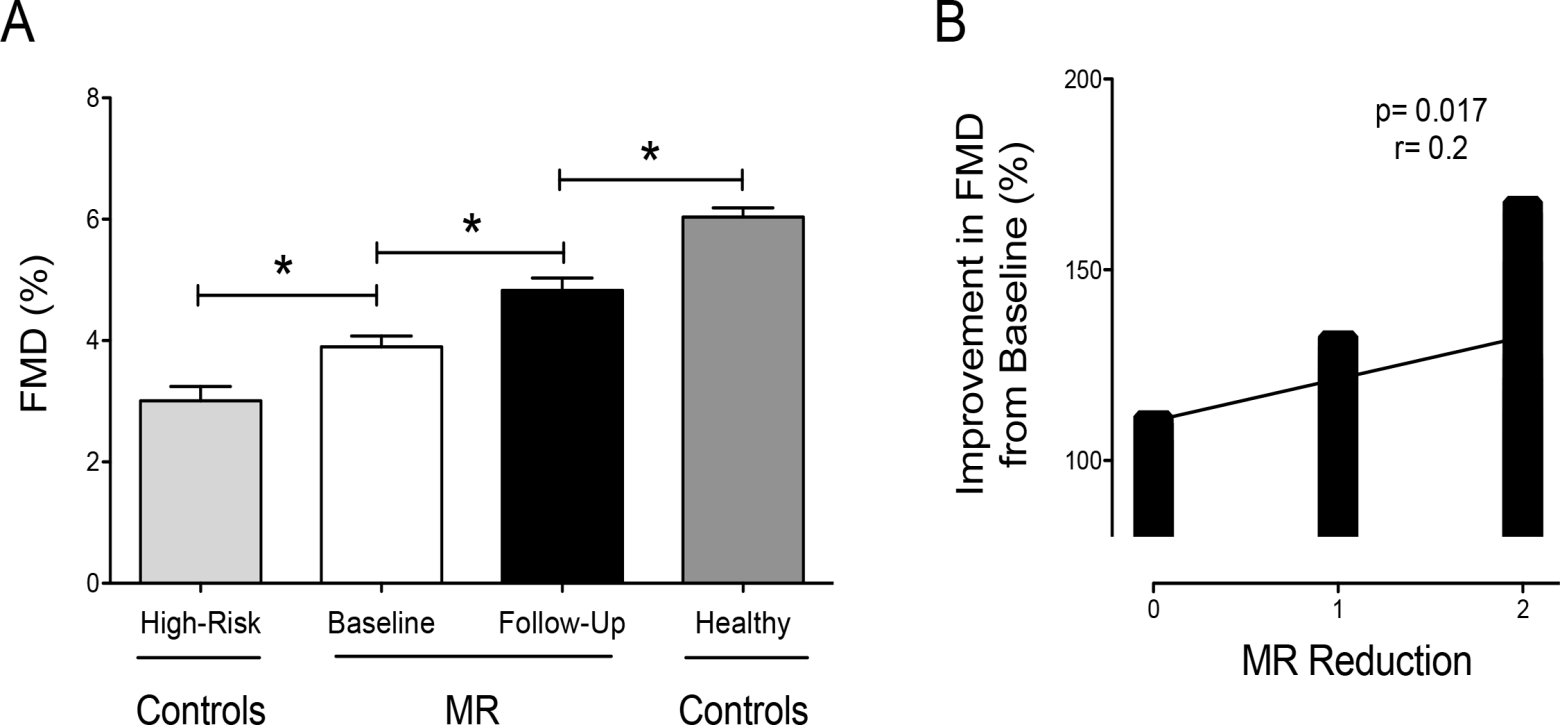
FMD (Flow-mediated dilation) in comparison to controls and the association with the degree of MR reduction. (A) FMD in patients with mitral regurgitation (MR) high-risk controls and healthy controls (* denotes p< 0.05, ANOVA with Bonferoni correction). (B) The degree of mitral regurgitation reduction is associated with improvement in FMD. MR reduction was graded as slight (0), moderate (1) or high reduction (2). Improvement in FMD is given as percent change from baseline.

Comparison of FMD values shows diminished levels in high-risk compared to MR patients at baseline. An increased endothelial function is observed in MR patients treated with PMVR and the highest levels are found in age-matched healthy controls (Fig 4A). Moreover, the improvement in FMD was associated with the degree of MR reduction (Fig 4B).

## Discussion

The major findings of this study are that i) patients with MR demonstrate significant levels of cell free Hb along with reduced endothelial function, ii) PMVR improves clinical status and ventricular function, and iii) leads to decreased Hb decompartmentalization with improvements in vascular endothelial function after three months.

Endothelial dysfunction is the proceeding factor and also the facilitative process for establishment of CVD. Importantly, the development of MR is associated with a reduced prognosis, regardless of its etiology as seen in patients with HF [10, 31]. We now reveal that patients with severe MR exhibit impaired vascular function. This is in line with a phenomenon using a dog model with genetically predisposed MR development. It was shown that mitral valve disease *per se* is associated with declined vascular endothelial functions [32]. Moreover, in patients with MR increased numbers of plasma endothelial microparticles have been demonstrated, leading to a reduction in mitral valve’s endothelial function. This is associated with the inhibition of the Akt/eNOS-HSP90 signaling pathway [33].

Our results give evidence that MR is associated with the release of Hb, which is able to limit NO bioavailability, as demonstrated in hemodialysis patients by our group [8]. The sufficient diffusional gradient for NO between the endothelium and the smooth muscle is impaired by the NO scavenging activity of Hb [34]. Excess NO scavenging by cell-free Hb has been shown to disturb the physiological vasodilation [9]. We have previously shown that nearly 70% of the measurable Hb-related heme is competent to consume NO [8]. NO reacts rapidly with Hb in a reaction producing nitrate and methemoglobin. It is thus tempting to speculate that NO activates the guanylyl cyclase and triggers vascular smooth muscle vasodilation mainly when hemoglobin is compartmentalized within red blood cells [7]. Corroborating this, it was shown that only small amounts of cell-free Hb are necessary to counterweigh smooth muscle available NO and to result in endothelial dysfunction [35].

It is known that impaired vasodilator function plays an important role in the initiation and progression of HF, being associated with adverse outcomes [2, 36]. There is increasing evidence suggesting that these abnormal cardiovascular phenotypes characterizing HF are triggered by impaired NO homeostasis [37–39]. This in turn propagates development and progression of HF [40, 41]. We now provide first evidence that a structural approach that improves HF symptoms through reduction of MR severity reduces Hb decompartmentalization and improves endothelial functions. While the improvement in endothelial function was additionally associated with the degree of MR reduction, p values should not be overinterpreted due to small sample size. PMVR has evolved as a key therapeutic option for patients with severe and moderate-to-severe MR who are at high risk and declined for surgery [42–44]. Recently it was shown, that PMVR is feasible in patients with end-stage HF, entailing clinical benefits [45]. While our patients were characterized by only moderate reduced LV-EF, it would be of interest to elucidate the benefits of PMVR on vascular endothelial functions in patients with marked LV dysfunction.

The inherent limitation of our study is the limited sample size. Due to the real-world setting and design of our study, we did not intend to preselect individuals with HF-related MR without vascular disease. Thus, we cannot exclude that the reduced endothelial function at baseline is related to the abundant comorbidities in our study group. Due to differences in pathophysiological origin of primary and secondary MR, further studies investigating these different disease entities and impact of PMVR on vascular functions should be performed. Finally, we cannot rule out the possibility that improvements in endothelial function are also mediated by improvement of ventricular function as observed following PMVR.

Our findings contribute to the understanding of PMVR-related improvement of cardiovascular functions. The impact of enhanced NO bioavailability has to be further investigated in order to determine whether reduction in cell-free HB and improvement in FMD could be used to predict responders vs. non-responders in patients undergoing PMVR.

## Supporting Information

S1 Table Basic clinical and biochemical characteristics of healthy controls and high-risk controls. (DOCX)
