# Supplementary material for: Percutaneous Mitral Valve Repair in Mitral Regurgitation Reduces Cell-Free Hemoglobin and Improves Endothelial Function
Source: PLoS One. 2016 Mar 17;11(3):e0151203. doi: 10.1371/journal.pone.0151203 (PMC4795750; doi:10.1371/journal.pone.0151203)
Supplement: S1 Table — (DOCX) [file pone.0151203.s001.docx]

## S1 Table. Basic clinical and biochemical characteristics of healthy controls and high-risk controls.

|  |  | **Healthy Controls**  **n=20** | **High-risk Controls**  **n=12** |
| --- | --- | --- | --- |
|  | Age (y) | 62,9 ± 5,2 | 67,9 ± 6,7 |
|  | Gender m (n) | 14 | 10 |
|  | Height (cm) | 175,5 ± 10,3 | 171,1 ±10,2 |
|  | Weight (kg) | 80,1 ± 16,6 | 88,6 ± 18 |
|  | BMI (kg/m^2^) | 25,5 ± 2,8 | 30,3 ± 5,9 |
|  | Smoker (n) | 2 | 3 |
|  | NYHA 3 (n) | 0 | 0 |
|  | NYHA 4 (n) | 0 | 0 |
|  | Logistic Euro-Score |  |  |
|  | Eurosscore II |  |  |
|  | Primary MR | 0 | 0 |
|  | Secondary MR | 0 | 0 |
| **Comorbidities** | |  |  |
|  | Diabetes (n) | 0 | 7 |
|  | Hypertension (n) | 0 | 12 |
|  | Pulmonary disease (n) | 0 | 3 |
|  | Peripheral artery disease (n) | 0 | 4 |
|  | pHT (n) | 0 | 0 |
|  | CAD (n) | 0 | 12 |
|  | Persistent atrial fibrillation (n) | 0 | 2 |
|  | Hyperlipoproteinemia (n) | 0 | 10 |
|  | CABG (n) | 0 | 0 |
|  | Pacemaker (n) | 0 | 0 |
| **Previous Interventions** | |  |  |
|  | History of CABG (n) | 0 | 0 |
|  | History of valv. intervention (n) | 0 | 0 |
|  | History of AMI (n) | 0 | 0 |
| **Medications** | |  |  |
|  | ACE-I/ARB (n) | 0 | 6 |
|  | Aldosterone-Antagonist (n) | 0 | 0 |
|  | Beta blocker (n) | 0 | 11 |
|  | Diuretics (n) | 0 | 10 |
|  | Anticoagulation (n) | 0 | 2 |
| **Clinical routine** | |  |  |
|  | GFR (ml/min) | 89,9 ± 11,7 | 8,2 ± 3,5 |
|  | Hb (g/dl) | 14,3 ± 1,0 | 11,1 ± 1,0 |
|  | Trop (mg/dl) | 6,7 ± 2,6 | 59,6 ± 25,3 |
|  | BNP (pg/ml) | 83,2 ± 55,1 | 12271 ±13240 |
|  | CRP (mg/dl) | 0,4 ± 0,1 | 1,29 ± 2,4 |
|  | Total Protein (g/dl) | 7,1 ± 0,3 | 7,0 ± 0,6 |
